# Tolvaptan use in children and adolescents with autosomal dominant polycystic kidney disease: rationale and design of a two-part, randomized, double-blind, placebo-controlled trial

**DOI:** 10.1007/s00431-019-03384-x

**Published:** 2019-05-03

**Authors:** Franz Schaefer, Djalila Mekahli, Francesco Emma, Rodney D. Gilbert, Detlef Bockenhauer, Melissa A. Cadnapaphornchai, Lily Shi, Ann Dandurand, Kimberly Sikes, Susan E. Shoaf

**Affiliations:** 1 Division of Pediatric Nephrology, University Children’s Hospital Heidelberg, Im Neuenheimer Feld 430, 69120 Heidelberg, Germany; 2 Department of Pediatric Nephrology, University Hospitals Leuven, Herestraat 49, B-3000 Leuven, Belgium; 3 PKD Research Group, Department of Development and Regeneration, KU Leuven, Herestraat 49, B-3000 Leuven, Belgium; 4 Pediatric Nephrology, Bambino Gesù Children’s Hospital, Piazza Sant’ Onofrio 4, 00165 Rome, Italy; 5 Regional Paediatric Nephro-Urology Unit, Southampton Children’s Hospital, Tremona Road, Southampton, SO16 6YD UK; 6 UCL Department of Renal Medicine, UCL Medical School, Rowland Hill Street, London, NW3 2PF UK; 7 Department of Paediatric Nephrology, Great Ormond Street Hospital for Children NHS Foundation Trust, Great Ormond Street, London, WC1N 3JH UK; 8 Rocky Mountain Pediatric Kidney Center, Rocky Mountain Hospital for Children at Presbyterian/St. Luke’s Medical Center, 2055 High Street, Suite 270, Denver, CO 80205 USA; 9 Otsuka Pharmaceutical Development & Commercialization, 2440 Research Boulevard, Rockville, MD 20850 USA; 10 Otsuka Pharmaceutical Development & Commercialization, 508 Carnegie Center Drive, Princeton, NJ 08540 USA

**Keywords:** Nephrology, Clinical trial, Autosomal dominant polycystic kidney disease, Tolvaptan, Pharmacotherapy

## Abstract

This report describes the rationale and design of a study assessing tolvaptan in children with autosomal dominant polycystic kidney disease (ADPKD). Phase A is a 1-year, randomized, double-blind, placebo-controlled, multicenter trial. Phase B is a 2-year, open-label extension. The target population is at least 60 children aged 12–17 years, diagnosed by family history and/or genetic criteria and the presence of ≥ 10 renal cysts, each ≥ 0.5 cm on magnetic resonance imaging. Subjects will be allocated into 4 groups: females 15–17 years; females 12–14 years; males 15–17 years; and males 12–14 years. Up to 40 subjects aged 4–11 years may also enroll, provided they meet the entry criteria. Weight-adjusted tolvaptan doses, titrated once to achieve a tolerated maintenance dose, and matching placebo will be administered twice-daily. Assessments include spot urine osmolality and specific gravity (co-primary endpoints), height-adjusted total kidney volume, estimated glomerular filtration rate, pharmacodynamic parameters (urine volume, fluid intake and fluid balance, serum sodium, serum creatinine, free water clearance), pharmacokinetic parameters, safety (aquaretic adverse events, changes from baseline in creatinine, vital signs, laboratory values including liver function tests), and generic pediatric quality of life assessments.

*Conclusion*: This will be the first clinical study to evaluate tolvaptan in pediatric ADPKD.

**Table:** 

**What is Known:** *• Autosomal dominant polycystic kidney disease (ADPKD) is a genetic disorder causing the development of cysts that impede kidney function over time and eventually induce renal failure* *• There are few data on the effects of tolvaptan, the only treatment approved for adults to slow disease progression, in pediatric ADPKD patients with early-stage disease*	
**What is New:** *• A phase 3, placebo-controlled study is evaluating tolvaptan over 3 years in children and adolescents with ADPKD* *• This study is designed to account for challenges of tolvaptan dosing and outcome assessment specific to the pediatric population*

**What is Known:**

*• Autosomal dominant polycystic kidney disease (ADPKD) is a genetic disorder causing the development of cysts that impede kidney function over time and eventually induce renal failure*

*• There are few data on the effects of tolvaptan, the only treatment approved for adults to slow disease progression, in pediatric ADPKD patients with early-stage disease*

**What is New:**

*• A phase 3, placebo-controlled study is evaluating tolvaptan over 3 years in children and adolescents with ADPKD*

*• This study is designed to account for challenges of tolvaptan dosing and outcome assessment specific to the pediatric population*

## Introduction

Autosomal dominant polycystic kidney disease (ADPKD) is the most common inherited disorder of the kidney [8, 18]. Dysregulation of the primary cilium on renal epithelial cells causes localized and unregulated expansion of the nephronic epithelium, resulting in the formation of fluid-filled cysts that grow and ultimately obstruct nephrons, blood vessels, and lymphatics [10]. In general, disease progression is inexorable, with an estimated 45–70% of patients developing end-stage kidney disease (ESKD) by age 65 years [11]. ADPKD is the fourth most common cause of ESKD, after diabetes, hypertension, and glomerulonephritis, accounting for 10% of patients receiving dialysis or kidney transplantation in Europe [17].

Tolvaptan, an oral selective antagonist of the vasopressin V2 receptor, is the only medication for ADPKD that has demonstrated beneficial disease-modifying properties in adults. Phase 3 clinical trials demonstrated the efficacy of tolvaptan in slowing the decline of kidney function in adults with relatively early-stage ADPKD and high likelihood of rapid disease progression (the Tolvaptan Efficacy and Safety in Management of Autosomal Dominant Polycystic Kidney Disease and Its Outcomes 3:4 [TEMPO 3:4] trial) and those with more advanced disease (the Replicating Evidence of Preserved Renal Function: an Investigation of Tolvaptan Safety and Efficacy in ADPKD [REPRISE] trial) [19, 21].

The Consortium for Radiologic Imaging Studies of Polycystic Kidney Disease (CRISP) established that kidney enlargement is a continuous process in ADPKD, with cysts expanding in young patients years or even decades before renal damage becomes apparent [1, 9, 14]. Cysts steadily develop and grow in children with ADPKD while kidney function remains normal, even as these patients manifest early symptoms such as nocturnal hypertension [12]. It is therefore reasonable to hypothesize that treatment with a disease-modifying agent like tolvaptan prior to adulthood might have beneficial effects on long-term outcomes. Of relevance, a recent case study found that treatment with tolvaptan resolved hyponatremia and prevented kidney enlargement in a female infant with confirmed ADPKD [7]. To further address the potential role of tolvaptan in pediatric disease, the current report describes the rationale and design of the first study to assess the pharmacodynamics (PD), pharmacokinetics (PK), safety, and efficacy of tolvaptan in children and adolescents with ADPKD. The study design was developed, in discussions with regulatory authorities, to provide initial data on the use of tolvaptan in children with ADPKD at high risk of progression, with focus on safety of use, appropriate dosing, and pharmacodynamic activity.

## Methods

### Subjects

The target population will consist of at least 60 male and female subjects aged 12–17 years who have a diagnosis of ADPKD, as defined by the presence of renal cysts, ADPKD family history, and/or genetic criteria. To be eligible, subjects must have ≥ 10 renal cysts, each measuring ≥ 0.5 cm on magnetic resonance imaging (MRI) inspection. Prospective subjects who are MRI naive should have an ultrasound to confirm the presence of ≥ 4 cysts, each ≥ 1 cm in size, prior to receiving a trial-specific MRI inspection. Subjects aged 4–11 years will also be allowed to enter the study during the recruitment period for the target population, provided they meet entry criteria. For the younger group, MRI assessment is not performed; accordingly, the detection of ≥ 4 renal cysts on ultrasound, each ≥ 1 cm in size, suffices to confirm cyst presence. (Full inclusion/exclusion criteria in Table 1) While the required enrollment is 60 subjects aged 12–17 years, it is expected that the trial may also enroll approximately 40 subjects aged 4–11 years, for a total sample size of approximately 100 subjects.

**Table 1:** Eligibility requirements

Inclusion criteria	
• Male and female subjects aged 4 to 17 years (inclusive) with a diagnosis of ADPKD as defined by the presence of family history and/or genetic criteria AND who have at least 10 renal cysts, each of which measures at least 0.5 cm, confirmed upon MRI inspection; subjects under the age of 12 years must have at least 4 cysts that are at least 1 cm in size, confirmed by ultrasound	
• Weight ≥ 20 kg	
• eGFR ≥ 60 mL/min/1.73 m^2^ within 31 days prior to randomization (using the Schwartz formula, eGFR = 0.413 × height [cm]/serum creatinine mg/dL [15])	
• Independent in toileting	
• Trial-specific written informed consent obtained from a parent/guardian or legally acceptable representative, as applicable for local laws, at screening, prior to the initiation of any protocol-required procedures. In addition, the subject must provide age-appropriate informed assent at screening and must be able to understand that he or she can withdraw from the trial at any time	
• Ability to swallow a tablet^a^	
• Ability to commit to remain fully abstinent (periodic abstinence [e.g., calendar, ovulation, symptothermal, post-ovulation methods] or withdrawal are not acceptable methods of contraception) or use two approved methods of birth control during the trial and for 30 days following the last dose of study drug for sexually active females of childbearing potential	
Exclusion criteria	
• Females who are breastfeeding and/or who have a positive pregnancy test result prior to receiving study drug	
• Liver function tests, including AST and ALT, ≥ 1.5 × upper limit of normal	
• Nocturnal enuresis	
• Need for chronic diuretic use	
• Subjects with advanced diabetes (e.g., glycosylated hemoglobin > 7.5% and/or glycosuria by dipstick, significant proteinuria, retinopathy), evidence of additional significant renal disease(s) (i.e., currently active glomerular nephritides), renal cancer, single kidney, or recent (within last 6 months) renal surgery or acute kidney injury	
• Subjects who have known clinically significant allergic reactions to chemicals with structure similar to tolvaptan (i.e., benzazepines): benzazepril, conivaptan, fenoldopam mesylate, or mirtazapine	
• Subjects having disorders in thirst recognition or inability to access fluids	
• Subjects who have bladder dysfunction and/or difficulty voiding	
• Subjects with critical electrolyte imbalances, as determined by the investigator	
• Subjects with or at risk of significant hypovolemia, as determined by investigator	
• Subjects with a history of substance abuse (within the last 6 months)	
• Subjects 12 years of age and older having contraindications to, or interference with, MRI assessments (e.g., ferro-magnetic prostheses, aneurysm clips, severe claustrophobia)	
• Subjects taking a vasopressin agonist (e.g., desmopressin)	
• Subjects with a history of persistent noncompliance with antihypertensive or other important medical therapy	
• Subjects taking medications or having concomitant illnesses likely to confound endpoint assessments, including taking approved (i.e., marketed) therapies for the purpose of affecting polycystic kidney disease cysts such as tolvaptan, vasopressin antagonists, anti-sense RNA therapies, rapamycin, sirolimus, everolimus, or somatostatin analogs (i.e., octreotide, sandostatin)	
• Has any medical condition that, in the opinion of the investigator, could interfere with evaluation of the trial objectives or safety of the subjects	
• Is deemed unsuitable for trial participation in the opinion of the investigator	
• Subjects who received any investigational agent in a clinical trial within 30 days prior to screening	
• Subjects who have a known lactose intolerance	
• Subjects who have had cyst reduction surgery within 6 weeks of the screening visit	

*ADPKD* autosomal dominant polycystic kidney disease, *ALT* alanine aminotransferase, *AST* aspartate aminotransferase, *eGFR* estimated glomerular filtration rate, *MRI* magnetic resonance imaging, *RNA* ribonucleic acid

^a^Must also meet health authority/ethics committee age restrictions on tablet use (if applicable)

### Study design

This is an ongoing, phase 3b, two-part study (EudraCT number: 2016-000187-42; ClinicalTrials.gov identifier: NCT02964273) [3, 5]. Enrollment started in September 2016, and the estimated primary completion date is December 2019, with study completion anticipated in December 2021 [3]. Twenty study sites located in Belgium, Germany, Italy, and the UK are participating. The trial is being conducted in compliance with FDA regulations, International Conference on Harmonization Good Clinical Practice Guideline (E6), international ethical principles derived from the Declaration of Helsinki, Council for International Organizations of Medical Science guidelines, and applicable local laws and regulations. Age-appropriate assent documents were created.

#### Phase A

**Fig. 1:**
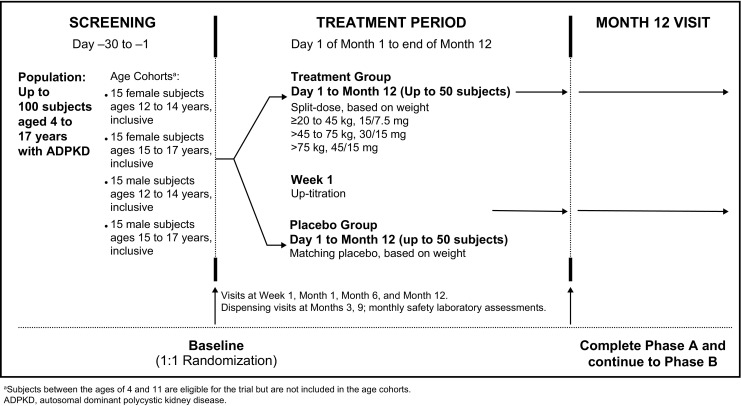
Overall study design of phase A (randomized, double-blind). ^a^Subjects between the ages of 4 and 11 are eligible for the trial but are not included in the age cohorts. ADPKD, autosomal dominant polycystic kidney disease

Phase A is a 1-year, randomized, double-blind, placebo-controlled, multicenter trial (Fig. 1). The target population consists of four cohorts (*n* = 15 per cohort), stratified by gender and age: males and females 15–17 years and males and females 12–14 years. Following double-blind 1:1 randomization, eligible subjects will receive either tolvaptan, administered as 7.5-, 15-, and 30-mg immediate-release tablets, or matching placebo tablets for up to 12 months. Tolvaptan and placebo will be administered every day as split-dose regimens, with the first dose taken upon waking and the second taken 8–9 h later. Each dose will be administered with a recommended 240 mL of water consumed within a 1-h period. Subjects are additionally encouraged to drink plain water per thirst throughout the day and one to two glasses of water before bedtime to help maintain proper hydration status.

Starting doses are based on weight. After 1 week, subjects who tolerate their initial dose will up-titrate once, as noted in Table 2. Subjects may down-titrate at any time in order to determine the range of tolerated doses.

**Table 2:** Tolvaptan weight-based dosing

Body weight	Starting dose regimen	Up-titrated dose regimen
≥ 20 kg to < 45 kg	15/7.5 mg tolvaptan or matching placebo	30/15 mg
≥ 45 kg to ≤ 75 kg	30/15 mg tolvaptan or matching placebo	45/15 mg
> 75 kg	45/15 mg tolvaptan or matching placebo	60/30 mg

#### Phase B

**Fig. 2:**
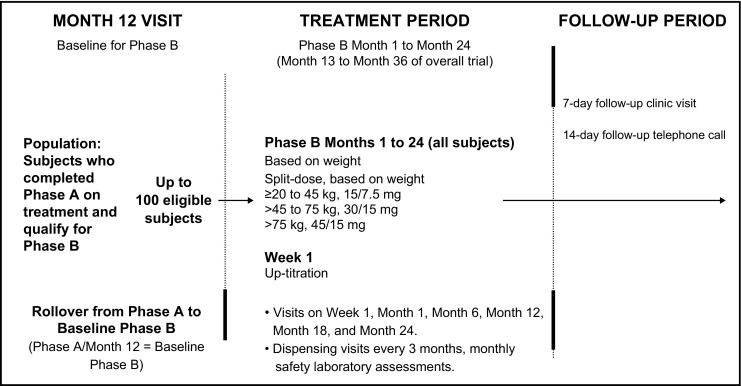
Overall study design of phase B (open-label extension)

Phase B is a 2-year, open-label extension in up to 100 subjects aged 4–17 years who completed phase A on treatment, are willing to continue in the study, and do not have any adverse events (AEs) requiring study drug discontinuation (Fig. 2).

.

### Efficacy and pharmacodynamic assessments

#### Phase A

In phase A, spot urine osmolality and spot urine specific gravity (co-primary endpoints) will be assessed at baseline, week 1, and month 1. Serum creatinine will be assessed at all visits, and serum sodium will be assessed at all visits until month 11. The number of daytime and nighttime voids will be recorded at week 1, month 1, month 6, and month 12, and 24-h fluid balance will be evaluated at week 1.

Subjects 12–17 years of age will have their kidneys scanned by MRI according to published standards [19] at screening and month 12. All subjects will have renal pelvic measurements by ultrasound at screening, month 1, and month 12. Subsequent measurement of kidney dimensions and evaluation of height-adjusted TKV will be performed at a central laboratory by individuals blinded to treatment assignment. As treatment effects on TKV or kidney function may be difficult to discern in a pediatric population with early-stage disease, pharmacodynamic parameters were considered the most appropriate co-primary endpoints for this study. Percent change from baseline to month 12 in height-adjusted TKV was designated as the key secondary endpoint. All primary, secondary, and exploratory endpoints of the trial, for both the 12–17- and the 4–11-year-old age groups, are listed in Table 3.

**Table 3:** Endpoints of the study

Co-primary endpoints (phase A)	
• Change from baseline in spot urine osmolality (premorning dose) after 1 week of daily dosing	
• Change from baseline in specific gravity (premorning dose) after 1 week of daily dosing	
Key secondary endpoint	
• Percent change in htTKV from phase A baseline to month 12, as measured by MRI	
Other secondary endpoints	
• 24-h fluid balance prior to week 1 in phase A	
• Change from baseline in renal function (eGFR by Schwartz formula [15]) at each clinic visit (week 1, month 1, month 6, and month 12 in phase A)	
• Change from baseline in renal function (eGFR by Schwartz formula) at each clinic visit (week 1, month 1, month 6, month 12, month 18, and month 24 in phase B)	
• Percent change in htTKV as measured by MRI from phase B baseline to phase B month 12	
• Percent change in htTKV as measured by MRI from phase B baseline to phase B month 24	
• Pharmacodynamic endpoints of urine volume (including 24-h fluid volume), fluid intake and fluid balance, sodium, creatinine, and free water clearance during dense PK sampling (after at least 1 month on study drug)	
• Proportions of each Tanner stage by gender and age compared to normative populations at baseline, month 6, and month 12 during the placebo-controlled phase (phase A), and every 6 months during the open-label extension phase (phase B)	
• Description of changes from baseline percentiles for height and weight by gender and age at baseline, month 6, and month 12 during the placebo-controlled phase (phase A), and every 6 months during the open-label extension phase (phase B)	
• Safety variables (changes from baseline in creatinine, vital signs, laboratory values including liver function tests, rate of aquaretic adverse events) in placebo and tolvaptan	
Exploratory endpoints	
• Percent change in htTKV as measured by MRI from phase A baseline to phase B month 24	
• Change from phase A baseline in spot urine osmolality (premorning dose) and specific gravity (premorning dose) after 1 month (phase A only)	
• Time to discontinuation due to any reasons in phase A and phase B	
• Tolvaptan maximum (peak) plasma concentration (*C*_max_), minimum plasma concentration (*C*_min_), time to *C*_max_ (*t*_max_), and area under the concentration-time curve from time zero to 24 h (AUC_0–24 h_) following dense PK sampling	
• PK sampling for separate population analysis	
• Tolvaptan metabolite concentrations from dense sampling	
• Generic pediatric quality of life assessments	
• Daytime and nighttime void collection	
Endpoints for subjects < 12 years who have ultrasound assessments	
• Percent change in htTKV as measured by ultrasound from phase A baseline to phase A month 12	
• Percent change in htTKV as measured by ultrasound from phase A baseline to phase B month 24	
• Percent change in htTKV as measured by ultrasound from phase B baseline to phase B month 24	
• Percent change in htTKV as measured by ultrasound from phase B baseline to phase B month 12	

*eGFR* estimated glomerular filtration rate, *htTKV* height-adjusted total kidney volume, *MRI* magnetic resonance imaging, *PK* pharmacokinetic

#### Phase B

In phase B, serum creatinine and serum sodium assessments will occur at all visits until month 24. Twenty-four-hour fluid balance will be evaluated at week 1. MRI scans of the kidney will occur at month 12 and month 24. Renal pelvic measurements by ultrasound will occur at month 1 and month 12.

### Pharmacokinetic assessments

During phase A only, sparse PK blood samples will be collected from all subjects at week 1, month 1, month 6, and month 12 for determination of steady-state concentrations of plasma tolvaptan and metabolites.

In addition, after at least 1 month on treatment during phase A, a subset of 20 consenting subjects in the 12–17-year-old age group (10 on tolvaptan and 10 on placebo) will undergo dense PK and PD sampling for 24 h following the schedule and procedures described by Shoaf et al. [16]. The blind in phase A will be maintained in this dense PK subpopulation via an Interactive Response System [13].

### Subject-reported outcome assessments

Subject-reported outcomes are assessed using generic pediatric quality-of-life questionnaires and information relevant to the medical, social, and economic consequences of new and ongoing ADPKD-related morbidities.

### Safety assessments

Safety is assessed throughout the study by AE assessments, clinical laboratory results, physical examination, vital signs, and 12-lead electrocardiogram. At each study visit, the investigator elicits AEs with a nonleading question, and all AEs reported by a subject must be recorded. Subjects in this study will be tested for alanine aminotransferase (ALT), aspartate aminotransferase (AST), alkaline phosphatase (ALP), and total bilirubin (BT) at screening/baseline and for ALT and AST at week 1 and every month for the duration of the study. The appearance of any suspicious symptom or sign in the investigator’s clinical judgment (a liver function test 2 × upper limit of normal [ULN] is generally accepted as a clinically significant occurrence across various medical disciplines) will trigger prompt re-testing, i.e., within 48–72 h. If significant abnormalities in transaminases and/or bilirubin are confirmed, other testing to fully evaluate the likely proximate cause will be undertaken, with the option of consulting local hepatic experts for interpretation and guidance. In addition, a hepatic adjudication committee has been convened for the purpose of oversight of hepatic events.

Liver transaminase or bilirubin levels reaching or exceeding 2 × ULN that have an uncertain or rapidly increasing trajectory will prompt at least temporary interruption of tolvaptan. Following interruption, tolvaptan will not be resumed until monitoring indicates abnormalities have resolved, are stable, or are not rapidly increasing, and then only with an increased frequency of monitoring (e.g., once weekly until returning to monthly monitoring at the discretion of the investigator) and if deemed appropriate in the clinical judgment of the investigator. A subject will be discontinued from the study on confirmation of any of the following criteria: ALT or AST ≥ 8 × ULN; ALT or AST ≥ 5 × ULN for more than 2 weeks; ALT or AST ≥ 3 × ULN and either BT ≥ 2 × ULN or international normalized ratio > 5; and ALT or AST ≥ 3 × ULN with appearance of fatigue, nausea, vomiting, right upper quadrant pain or tenderness, fever, rash, and/or eosinophilia (> 5%) and signs of jaundice.

### Statistics

Phase A will have two co-primary endpoints: change from baseline in spot urine osmolality and specific gravity (premorning dose) after 1 week of daily dosing. For the primary endpoint analysis, a sample size of at least 60 subjects aged 12–17 years inclusive is expected. Since data will be summarized using descriptive statistics and are aimed at testing safety and tolerability, no formal power calculations were undertaken.

Efficacy assessments will be conducted on the full analysis set, defined as subjects who have been randomized to a treatment group, have received at least one dose of study drug, and have both a phase A baseline and at least one post-baseline efficacy evaluation. Subgroup analyses of the primary and key secondary endpoints will be performed to examine differences in treatment response based on phase A baseline status (e.g., gender, age stratum). For all secondary endpoints, descriptive statistics by visit will be presented.

Safety analyses will be conducted on all subjects who received one dose of study drug. Summary statistics of changes from phase A and phase B baseline will be provided for safety variables based on all available data.

## Discussion

The purpose of the study described here is to assess the safety, tolerability, PD, PK, and efficacy of tolvaptan in children with ADPKD. As this will be the first study to assess tolvaptan in pediatric subjects with ADPKD, a number of features associated with the study population have been considered and addressed. For example, it is expected that very few, if any, of the screened participants will present with disease-specific symptoms, and formal diagnostic criteria for ADPKD so early in disease progression have yet to be developed. To circumvent this issue, an advisory panel of experts was convened to develop screening standards. The consensus opinion was that family history/genetics, cyst number as assessed by MRI (or ultrasound in younger subjects), and progression of disease that would warrant treatment are together the most reasonable approach to diagnose early disease. Therefore, to be eligible for enrollment, subjects must have a family history and/or genetic criteria for ADPKD and ≥ 10 renal cysts, each measuring ≥ 0.5 cm on MRI; subjects under the age of 12 years must have ≥ 4 cysts, each measuring ≥ 1 cm on ultrasound.

The early stage of polycystic kidney disease in the pediatric study population also complicates efficacy assessments. Post hoc analyses of TEMPO 3:4 showed that the beneficial treatment effects of tolvaptan on TKV and eGFR were similar across chronic kidney disease (CKD) stages, including among subjects early in disease progression (CKD 1) [20]. Although clinically significant, the absolute changes in TKV growth and eGFR decline versus placebo were nonetheless relatively small across CKD stages and could be particularly difficult to detect in pediatric subjects. Given that a large sample size and long follow-up would likely be necessary to discern treatment effects on TKV growth and eGFR decline in a pediatric population, the study steering committee and regulatory authorities agreed that pharmacodynamic effects would be more practicable and informative primary study endpoints in a first trial of tolvaptan for the treatment of pediatric ADPKD. Accordingly, the co-primary endpoints are change from baseline in spot urine osmolality and in urine specific gravity. These endpoints will assess whether tolvaptan acts in the same way in children/adolescents as it does in adults, which has not been examined before. Results from these analyses should inform the choice of assessments in future pediatric trials.

In adults, optimal ADPKD treatment requires constant 24-h inhibition of the V2 receptor, and patients are thus typically encouraged to take maximally tolerated doses. Adults receive a split-dosing regimen to maintain suppression of vasopressin action across 24 h, consisting of a higher dose early in the day, followed by a lower dose approximately 8–9 h later. The primary tolerability concern with tolvaptan dosing is excretion of solute-free water, or aquaresis, which manifests as polyuria (production of large volumes of dilute urine), polydipsia (excess thirst and drinking), nocturia (need to wake up to urinate at night), and pollakiuria (abnormally frequent urination) [19]. Post hoc analyses of the TEMPO 3:4 study population showed that aquaretic AEs resulting in tolvaptan discontinuation were more likely in subjects with higher baseline renal function [4]. Furthermore, sensitivity to aquaretic effects appeared to be highest during initial titration of the drug [4].

Combined, the adult data on aquaresis suggest that the current study’s pediatric ADPKD population, with its high renal clearance capacity and younger age demographic, could be especially sensitive to aquaretic AEs, particularly early during titration. Subjects within each cohort will therefore initiate treatment at a weight-adjusted split starting dose that is no greater than 67% of the maximal weight-adjusted starting dose for the adult subjects in TEMPO 3:4. This low starting dose will be administered for 1 week to allow acclimation to the excess diuresis commonly associated with tolvaptan and, hopefully, to avoid potential problems with aquaretic AEs early in titration. Only after 1 week at the starting dose, if tolerated, will the dosage be up-titrated once to the weight-adjusted starting dose used in TEMPO 3:4. As is the case with adult dosing, participants will receive a higher dose early in the day, followed by a lower dose administered approximately 8–9 h later, which produces maximal inhibition on waking and a gradual fall-off of effect during the night, when frequent urination would lead to interruption of sleep.

The most notable safety issue with tolvaptan in adult ADPKD studies was the potential for idiosyncratic hepatic toxicity [22]. To mitigate this risk, the frequency of liver function testing was increased from once every 4 months in TEMPO 3:4 to once monthly in REPRISE [22]. Accordingly, subjects in the current pediatric study will be tested for ALT/AST, ALP, and BT during screening and for ALT/AST at week 1 and monthly during the entire trial. Evidence of liver damage will be assessed by the local physician, an independent hepatic adjudication committee, and an independent data monitoring committee. In addition, stringent drug interruption rules will be employed to ensure rapid response to any potential hepatic signals.

The current study has some potential limitations resulting from its design. First, the dramatic difference in aquaretic effect between tolvaptan and placebo may lead the subject and/or investigator to perceive treatment assignment. The prescription of additional fluids to subjects during study drug administration may serve to confound this effect, and it should be noted that the pharmacodynamic and efficacy endpoints are objective and unlikely to be affected by the participant’s perceptions. Second, the study inclusion criteria relating to ADPKD diagnosis and cyst burden are largely opinion-based, and their predictive value for early disease progression remains unknown. The criteria are justifiable, however, given study data indicating that cyst burden correlates with the presence of hypertension in pediatric ADPKD patients, and that hypertension is predictive of rapid disease progression in this population [2, 6, 12]. Finally, tolerability of tolvaptan in a population that still has good renal function may be dose limiting or could cause early tolvaptan discontinuation. Nonetheless, the current study is expected to provide valuable information on the safety, tolerability, PD, PK, and efficacy of tolvaptan in pediatric patients with ADPKD and potentially inform the design of trials intended to assess therapeutic efficacy in this population.

## Abbreviations

ADPKD: Autosomal dominant polycystic kidney disease
AEs: Adverse events
ALP: Alkaline phosphatase
ALT: Alanine aminotransferase
AST: Aspartate aminotransferase
BT: Total bilirubin
CKD: Chronic kidney disease
CRISP: Consortium for Radiologic Imaging Studies of Polycystic Kidney Disease
DILI: Drug-induced liver injury
eGFR: Estimated glomerular filtration rate
ESKD: End-stage kidney disease
MRI: Magnetic resonance imaging
PD: Pharmacodynamics
PK: Pharmacokinetics
REPRISE: Replicating Evidence of Preserved Renal Function: an Investigation of Tolvaptan Safety and Efficacy in ADPKD
TEMPO 3:4: Tolvaptan Efficacy and Safety in Management of Autosomal Dominant Polycystic Kidney Disease and Its Outcomes 3:4
TKV: Total kidney volume
ULN: Upper limit of normal

## References

[1] Cadnapaphornchai MA (2015). Autosomal dominant polycystic kidney disease in children. Curr Opin Pediatr.

[2] Cadnapaphornchai MA, McFann K, Strain JD, Masoumi A, Schrier RW (2009). Prospective change in renal volume and function in children with ADPKD. Clin J Am Soc Nephrol.

[3] ClinicalTrials.gov (2019) Safety, pharmacokinetics, tolerability and efficacy of tolvaptan in children and adolescents with ADPKD (autosomal dominant polycystic kidney disease) [ClinicalTrials.gov identifier: NCT02964273]. https://clinicaltrials.gov/ct2/show/NCT02964273. Accessed 29 Mar 2019

[4] Devuyst O, Chapman AB, Shoaf SE, Czerwiec FS, Blais JD (2017). Tolerability of aquaretic-related symptoms following tolvaptan therapy in subjects with autosomal dominant polycystic kidney disease: results from TEMPO 3:4. Kidney Int Rep.

[5] EU Clinical Trials Register (2019) A phase 3b, two-part, multicenter, one year randomized, double-blind, placebo-controlled trial of the safety, pharmacokinetics, tolerability, and efficacy of tolvaptan followed by a two year open-label extension in children and adolescent subjects with autosomal dominant polycystic kidney disease (ADPKD) [EudraCT number: 2016-000187-42]. https://www.clinicaltrialsregister.eu/ctr-search/trial/2016-000187-42/GB/. Accessed 29 Mar 2019

[6] Fick-Brosnahan GM, Tran ZV, Johnson AM, Strain JD, Gabow PA (2001). Progression of autosomal-dominant polycystic kidney disease in children. Kidney Int.

[7] Gilbert RD, Evans H, Olalekan K, Nagra A, Haq MR, Griffiths M (2017). Tolvaptan treatment for severe neonatal autosomal-dominant polycystic kidney disease. Pediatr Nephrol.

[8] Grantham JJ (2008). Therapy for polycystic kidney disease? It’s water, stupid!. J Am Soc Nephrol.

[9] Grantham JJ, Chapman AB, Torres VE (2006). Volume progression in autosomal dominant polycystic kidney disease: the major factor determining clinical outcomes. Clin J Am Soc Nephrol.

[10] Grantham JJ, Mulamalla S, Swenson-Fields KI (2011). Why kidneys fail in autosomal dominant polycystic kidney disease. Nat Rev Nephrol.

[11] Lentine KL, Xiao H, Machnicki G, Gheorghian A, Schnitzler MA (2010). Renal function and healthcare costs in patients with polycystic kidney disease. Clin J Am Soc Nephrol.

[12] Massella L, Mekahli D, Paripovic D (2018). Prevalence of hypertension in children with early-stage ADPKD. Clin J Am Soc Nephrol.

[13] Ruikar V (2016). Interactive Voice/Web Response System in clinical research. Perspect Clin Res.

[14] Rule AD, Torres VE, Chapman AB (2006). Comparison of methods for determining renal function decline in early autosomal dominant polycystic kidney disease: the consortium of radiologic imaging studies of polycystic kidney disease cohort. J Am Soc Nephrol.

[15] Schwartz GJ, Munoz A, Schneider MF, Mak RH, Kaskel F, Warady BA, Furth SL (2009). New equations to estimate GFR in children with CKD. J Am Soc Nephrol.

[16] Shoaf SE, Chapman AB, Torres VE, Ouyang J, Czerwiec FS (2017). Pharmacokinetics and pharmacodynamics of tolvaptan in autosomal dominant polycystic kidney disease: phase 2 trials for dose selection in the pivotal phase 3 trial. J Clin Pharmacol.

[17] Spithoven EM, Kramer A, Meijer E, Orskov B, Wanner C, Abad JM, Areste N, Alonso de la Torre R, Caskey F, Couchoud C, Finne P, Heaf J, Hoitsma A, de Meester J, Pascual J, Postorino M, Ravani P, Zurriaga O, Jager KJ, Gansevoort RT, de los Angeles Garcia Bazaga M, Metcalfe W, Rodrigo E, Quiros JR, the EuroCYST Consortium, Budde K, Devuyst O, Ecder T, Eckardt KU, Gansevoort RT, Kottgen A, Ong AC, Petzold K, Pirson Y, Remuzzi G, Torra R, Sandford RN, Serra AL, Tesar V, Walz G, the WGIKD, Wuthrich RP, Antignac C, Bindels R, Chauveau D, Devuyst O, Emma F, Gansevoort RT, Maxwell PH, Ong AC, Remuzzi G, Ronco P, Schaefer F, on behalf of the ERA-EDTA Registry, ERA-EDTA Registry, EuroCYST Consortium, WGIKD (2014). Renal replacement therapy for autosomal dominant polycystic kidney disease (ADPKD) in Europe: prevalence and survival--an analysis of data from the ERA-EDTA Registry. Nephrol Dial Transplant.

[18] Torres VE, Harris PC, Pirson Y (2007). Autosomal dominant polycystic kidney disease. Lancet.

[19] Torres VE, Chapman AB, Devuyst O, Gansevoort RT, Grantham JJ, Higashihara E, Perrone RD, Krasa HB, Ouyang J, Czerwiec FS (2012). TEMPO 3:4 Trial Investigators (2012) Tolvaptan in patients with autosomal dominant polycystic kidney disease. N Engl J Med.

[20] Torres VE, Higashihara E, Devuyst O, Chapman AB, Gansevoort RT, Grantham JJ, Perrone RD, Ouyang J, Blais JD, Czerwiec FS, TEMPO 3:4 Trial Investigators (2016). Effect of tolvaptan in autosomal dominant polycystic kidney disease by CKD stage: results from the TEMPO 3:4 trial. Clin J Am Soc Nephrol.

[21] Torres VE, Chapman AB, Devuyst O (2017). Tolvaptan in later-stage autosomal dominant polycystic kidney disease. N Engl J Med.

[22] Watkins PB, Lewis JH, Kaplowitz N, Alpers DH, Blais JD, Smotzer DM, Krasa H, Ouyang J, Torres VE, Czerwiec FS, Zimmer CA (2015). Clinical pattern of tolvaptan-associated liver injury in subjects with autosomal dominant polycystic kidney disease: analysis of clinical trials database. Drug Saf.

